# Dental Disgust—An Ethnography of Abjection in Elderly Care

**DOI:** 10.1111/1467-9566.70061

**Published:** 2025-06-26

**Authors:** Louise Folker

**Affiliations:** ^1^ Department of Odontology Faculty of Health and Medical Sciences University of Copenhagen København Denmark; ^2^ Copenhagen Centre for Health Research in the Humanities (CoRe) Faculty of Humanities South Campus Saxo Institute University of Copenhagen København Denmark

**Keywords:** abjection, articulation, care, dental disgust, dirty work, disgust, elderly care, oral health

## Abstract

This study identifies dental disgust as a widespread experience among carers while caring for decayed and ill mouths, hindering vital daily dental care among care‐dependent older people. It is based on an ethnographic study conducted in nursing homes, home‐care units and a rehabilitation centre in Danish elderly care, and it suggests that dental disgust plays a profound role in the impairment of oral health in elderly care settings. The study demonstrates that dental disgust remains unacknowledged in care systems and care studies, leaving carers inappropriate/d, shameful and solely responsible for handling it. It explores dental disgust through Julia Kristeva's notion of abjection (1982) to illuminate some of its care‐hindering dynamics. It further draws on Donna Haraway's thoughts on articulation as a means of imagining better futures for people who have been depreciated as inappropriate (1992). Thereby, it suggests that the carers' articulations of dental disgust must be acknowledged and handled collectively. The study states that supporting carers institutionally and in research can help eliminate the inappropriateness of dental disgust, enabling future dental care. The study offers an analytical framework that transfers the theoretical terms ‘abjection’ and ‘articulation’ to socio‐materially situated settings and into practice‐relevant usage when disgust interferes with care.

Brushing older people’s teeth can make me feel sick to my stomach. Like (enacted vomiting sound and reflex movement). I feel so bad admitting it, but of course, I find it disgusting. We are not robots, after all.

(Aya, carer)

## 1 Introduction

The sensuous encounter with another person's mouth can be transgressive. The teeth may be broken, darkened or missing, while the mucosa may be swollen and filled with deposits. Saliva, mucus, food leftovers and vomit can abruptly or persistently intrude on the mouth and its surroundings. The mouth can be malodorous, and its odours can permeate entire intimate spheres, social interactions and interior environments. Alongside its vital significance for health, bodily processes and intimate and social life, the mouth can be a brutal, erratic and hard‐to‐access part of the body. Thus, when the mouth decays and oral diseases occur, dental care can become a crucial and difficult matter for carers in elderly care settings, encompassing various sensitive experiences for everyone involved.

This ethnographic study confronts the precarious, unclean and brutal parts of care work. It sheds light on the disgust provoked by dirty work (Twigg 2006) involving the abject body (Kristeva 1982). Not as a problem to be solved, rejected or judged but, rather, as a common and inevitable care condition requiring deliberate and collective handling. This study raises the following question: *How can good care be secured when disgust interferes with caring encounters?* As its case, the study explores how carers in the Danish elderly care system experience caring for ill and decayed mouths. It conceptualises and explores dental disgust. Based on long‐term fieldwork in Danish municipal elderly care, the study has found that nausea, vomiting reflexes, trembling and a sense of boundary violation regarding others' and personal boundaries make dental disgust a widespread experience among carers. Yet, despite its recurring everyday prevalence, the study concurrently found that dental disgust is greatly marginalised. It is deeply surrounded by inappropriateness, shame, and a lack of acknowledgement in care institutions. Therefore, it is most often kept secret by carers, who fear judgements and workplace repercussions. This is not only problematic for the carers, but dental disgust often means that care‐dependent older peoples' vital dental care is insufficiently performed, if it is even performed at all. This includes daily toothbrushing, the control of oral diseases and treatment access and adherence. The consequences are critical, as the failure to perform oral hygiene and untreated diseases can lead to social isolation, malnutrition, pneumonia and other severe diseases, especially late in life (Klotz et al. 2018; Lindmark et al. 2021). On this basis, this study suggests that dental disgust is significant in the impairment of oral health among care‐dependent older people (Petersen et al. 2021). The aim of the study is to understand dental disgust, dissolve its secretive shadows and openly approach it as a common care condition. Ultimately, to mitigate its harmful consequences for the sake of both carers and the cared‐for.

Studies within the field of odontology demonstrate that nondental healthcare professionals experience negative emotions (Johnson 2013), unpleasantness (Reed et al. 2006) and ‘a distaste of teeth and dentures’ (Frenkel 1999) when engaging with dental care and the mouth generally. Focussing on nurses in hospital settings, these studies report that they often find dental care discomforting, troublesome (Reed et al. 2006) and embarrassing to initiate and perform (Ohrn et al. 2000). Because these experiences often lead to avoidant behaviours among nurses, they are associated with inferior dental care being provided for patients (Furr et al. 2004). This study provides an ethnographic exploration of these aspects of the human mouth. It examines the concept of dental disgust and offers insights into its practices in a social–material context while critically and carefully providing knowledge about how to address it.

Disgust does not align with common conceptions of good care. In the nursing literature, disgust is described as a complex phenomenon involving the healthcare providers' responses to certain bodily symptoms and medical procedures, especially concerning infectious diseases (Ksiazkiewicz and Friesen 2020), body fluids (Burr 2009) and palliative care (Warmington et al. 2022), affecting well‐being, job satisfaction and staff turnover (Edgar et al. 2024). It is well documented that disgust reduces the quality of care work, risking negative patient outcomes (Adagide et al. 2024; Edgar et al. 2024; Hadjittofi et al. 2020). Recent psychological research demonstrates that nursing home carers often become habituated to disgust‐inducing care tasks over time. It suggests that carers can and often do become desensitised to disgust, even though it does not address whether this habituation translates into behavioural changes (Edgar et al. 2024). This study contributes to the field with an empirical investigation of how processes of disgust‐confrontation and its unconcealment can be supported collectively, in theory and in practice.

Care‐related disgust is closely linked to what sociologist Julia Twigg calls dirty work, or work ‘*involving what modern secular society does not want to think about: decay, dirt, death, decline, failure.*’ (Twigg 2000). Twigg explores how dirty work is devalued, contributing to the emotional load among carers, emphasising that it challenges not only the carers' work experiences but also how society perceives their labour. This resonates with sociologist Bill Hughes's post‐feminist ethics of care, which uses a waste discourse to demonstrate that the management of bodily exclusions, or ‘*what one does with one's shit, snot, sweat, saliva, sick, wind, blood and pee’,* creates social norms that can conflate the waste material with the wasted lives of the carers and cared for (Hughes et al. 2005, 266). Therefore, bodily care work is often associated with low pay, little or no prestige and traditionally female professions (Hughes et al. 2005; Twigg 2000). Against this background, this study suggests dental disgust as a multifaceted social issue encompassing various kinds of stigma and marginalisation.

As disgust is often left silenced in clinics and care settings (Kaiser et al. 2019; McCabe 2010), review studies call for more empirical research on care‐related disgust, including investigations of how it is handled in practice (Hadjittofi et al. 2020). Therefore, this study contributes empirical knowledge about disgust while insisting that it is socio‐materially situated, varying from praxis to praxis, and that its investigation, therefore, requires empirical sensitivity (Whyte 2009). The study further suggests that dental disgust is not problematic in and of itself, rather it becomes problematic because of the inappropriateness and marginalisation associated with it in care institutions, making it even more challenging for the individual carer. Therefore, this article does not describe dental disgust as a solely individual feature, a negative attitude (Eadie and Schou 1992) or an emotional phenomenon (Johnson 2013; Ohrn et al. 2000) that simply requires individualised coping strategies (Hadjittofi et al. 2020). Instead, it suggests that dental disgust and other kinds of care‐related disgust are also interpersonal, institutional, material, educational and work‐environmental phenomena and must be addressed as such.

## 2 Articulating Abjection—An Analytical Framework

This study explores dental disgust through the lens of Julia Kristeva's notion of abjection. In ‘Powers of Horror—An Essay on Abjection’ (1982), Kristeva emphasises abjection as the encounter with what we find disgusting, repulsive, defiling and monstrous—and thereby as a part of the human condition. Abjection is what reminds us of our mortality and bodily decay and what threatens the Western ideal of a clean and proper body. It provokes bodily reactions beyond our control; it leads to ‘*a gagging sensation and, still farther down, spasms in the stomach, the belly; and all the organs shrivel up the body, provoke tears and bile, increase heartbeat, cause forehead and hands to perspire*’ (1982, p. 2). Abjection is neither subject nor object; it is the encounter in‐between, challenging the boundaries between inner and outer worlds. ‘*It is thus not lack of cleanliness or health that causes abjection but what disturbs identity, system, order. What does not respect borders, positions, rules. The in‐between, the ambiguous, the composite*’ (1982, p. 4). In this approach, the subject of dental disgust is the carer; the object is the cared‐for mouth and abjection is their encounter; the dental care situation. On this note, abjection, when used as a lens, clarifies multifaceted dimensions of dental disgust to generate knowledge about what it comprises, including how it stands in the way of sufficient dental care in elderly care settings.

In this article, abjection is not merely a philosophical term. Instead, it is emphasised and utilised for its empirical relevance. The abjection lens is used to explore dental disgust as a situated and socio‐material phenomenon, as it highlights its various emotional, sensuous, material, technological, relational, institutional and structural aspects. According to Kristeva, abjection holds great transformative potential because it invites the subject to question its identity and construct new boundaries. ‘*I expel myself, I spit myself out, I abject myself within the same motion through which “I” claim to establish Myself*’ (1982, p. 3), Kristeva writes. Thereby, she calls attention to abjection because of the ‘*key position it assumes in the dynamics of the subject's constitution’* (1982, p. 47). Because abjection has the potential to transform the subject, it can transform what is being abjected. At its core, abjection is dynamic. However, for Kristeva, the transformation of abjection is a process that requires a confrontation with what provokes discomfort. Rather than outright rejection, Kristeva suggests that the subject must enter a complex relationship with the abject, ultimately reconstructing identity and the boundaries of the self. Therefore, Kristeva confidently writes, ‘*abject and abjection are my safeguards. The primers of my culture*’ (1982, p. 2). This study also utilises the abjection lens to illuminate the inherent transformational potential of dental disgust in practice. Thus, it not only aids in understanding dental disgust but also assists in its transformation and handling in praxis settings.

Within the field of social gerontology, Chris Gilleard and Paul Higgs reconceptualise abjection as a key term in ageing studies. They use the term ‘ageing abjection’ to understand cultural anxieties surrounding the fourth age (Gilleard and Higgs 2011), which is understood not as a chronological age but, as a social imaginary of old age associated with health decline and care needs (Gilleard and Higgs 2013). For Gilleard and Higgs, the re‐alignment of abjection arises from a perceived loss of bodily self‐control, self‐consciousness, social agency and from a destabilising of the subject in late life. ‘*What constitutes the core of abjection is not so much weakness or frailty but the evident failure of social intent, the inattention that betrays “self‐control” and “self‐direction”* (…) *in short, the impotence to mount a transgression of agedness*’ (2013, p. 139). Given this understanding, dental disgust is not about the teeth or mouth as such but about a lack of self‐control regarding dental care and its everyday practices, including toothbrushing, eating, drinking and smoking habits, as well as (economic) opportunities to see a dentist throughout (late) life. Furthermore, they discuss potential defences against ageing abjection, emphasising care work as a solution. They suggest that warm, close and tactile caring can redeem even profound abjection of the fourth age (Gilleard and Higgs 2011, 139). This article will challenge this emphasis on care as a solution to abjection. As the coming analysis will show, care is not a defence against abjection. When care is highlighted as a solution, it risks overlooking the precarity of bodily care and the carers’ concerns about further splintering the cared‐for subject and marginalising the disgusted carers even more, although disgust experiences seem to be quite common when working with ill, decayed and dying bodies. However, Gilleard and Higgs also raise a question about their conclusion. They ask whether care can always remedy age abjection or whether ‘*intimate and intense caring also risks dragging both carer and cared for deeper into abjection while removing any remaining scope for individual transgression’,* making care *‘a social sphere in which all participants are blighted because they live wasted lives*’ (Gilleard and Higgs 2011, 140). This is a crucial question, but they leave it unanswered and without further investigation. This point is exactly where this study begins. It suggests a new understanding of *how* care can redeem abjection, insisting that it requires open, collective and institutional handling. Therefore, the study investigates how care systems can secure a supportive framework of human and nonhuman actors when abjection interferes with care.

This study's analytical framework also draws on Donna Haraway's essay ‘The Promises of Monsters. A Regenerative Politics for Inappropriate/d Others’ (Haraway 1992). Here, Haraway reflects on how confronting the monstrous parts of existence can help do away with the marginalisation of people depreciated as inappropriate. Inspired by Haraway, this study suggests understanding carers who experience dental disgust as ‘*inappropriate/d others’* (1992) because of the sharp contrast with common conceptions of good care they face and because of the shame and secrecy that cling to their experiences. In Haraway's line of thought, inappropriateness is not an inherent feature but rather a social position people are placed in, which is why she formats the word *inappropriate/d.* (1992). Haraway delves into the idea of embracing the monstrous because it repeals rigid categories and dichotomies, such as fiction and fact or human and nature, and therefore serves as a catalyst for envisioning better futures for inappropriate/d others. She emphasises that embracing the monstrous can generate new significance. While reminding her reader that ‘*monsters have the same root as to demonstrate; monsters signify*’ (1992, p. 514), she writes that ‘*monsters generate distinctly diffracted views of the self, evident in beliefs and practices in relation to vulnerability and mortality*’ (1992, p. 499). On this basis, this study explores dental disgust as monstrous to reimagine better dental care situations in the future. Yet, monstrosity, here, is not only about marred, decayed and ill mouths. It is also about the cultural image of a carer being disgusted by a care‐dependent person, who is relying on and has the right to receive care.

According to Haraway, the reimagining of a better futures can occur through articulation: when the inappropriate/d others communicate its own meaning ‘*This world must always be articulated, from people's points of view’ and through “situated knowledge*”. (…) *To articulate is to signify. It is to put things together: scary things, risky things, contingent things. I want to live in an articulate world. We articulate; therefore, we are*’ (1992, p. 502). This study investigates some of the components revealed when dental disgust is viewed through the lens of abjection as articulation. Although the lens of abjection allows us to clarify the various components of dental disgust, Haraway's notion of articulation further allows an analysis of each emerging component. However, in Haraway's perspective, articulation extends beyond verbal expression or language. ‘*Articulation is not a simple matter’,* she writes, as they can have diverse forms of expression, encompassing words, sounds, silence, gestures, practices and uses of materials and technologies (1992, p. 501). Concurrently, this study seeks to dissolve the marginalising shadows surrounding dental disgust. The analytical framework thus serves as a two‐sided treatment for dental disgust: it confronts and transforms dental disgust while simultaneously abolishing the inappropriateness surrounding it. In other words, it combines and utilises the potential Kristeva identifies in abjection and the potential that Haraway identifies in articulation to discipline dental disgust and reimagine better dental care situations in the future.

## 3 Methods

This study originates from the *Lifelong Oral Health* research project, which is based at the Copenhagen Centre for Health Research in the Humanities (CoRe) and the Community Dentistry research group at the Department of Odontology, both of which are located at the University of Copenhagen. The project also involves two Danish municipalities and five of their elderly care units, encompassing two nursing homes, a rehabilitation centre and two outgoing home care units. The overall aim of the project is to identify barriers to oral health in Danish elderly care and develop initiatives to improve it. Disgust initially occurred at the very first project kick‐off workshop, as all project participants, including carers, were asked to write anonymous post‐its describing the barriers to oral health they experienced in their everyday lives. Several of these post‐its contained the words ‘*Gross*’, ‘*Transgressive*’, ‘*Big taboo*’ and ‘*Yuck*!’. Yet, it was challenging to have an open conversation about these issues with the carers at the beginning of the project. The initial fieldwork consisted of participant observations at the five units; semi‐structured interviews with older persons and relatives and focus group interviews with care workers, dental practitioners and local managers and six project seminars with all project partners. Clinical registrations of the oral health status of older people in the care units were conducted by dental researchers. During this initial fieldwork, the carers often shied away when asked about their personal experiences of performing dental care. Therefore, when trust between the research team and the carers emerged after working together on the project for 1.5 years, follow‐up fieldwork was initiated. Here, individual interviews with the carers were conducted to ensure a safe and anonymous interview environment. These consisted of semi‐structured interviews with 10 carers, two local managers and three municipal project managers, which were primarily conducted by the author of this article, who has a background in comparative literature and ethnology. Furthermore, two focus group interviews with four local managers and two double interviews with townhall managers were conducted by the research team. Most quotes and fieldnotes in this article originate from this follow‐up fieldwork.

All ethnographic material derived from the follow‐up fieldwork has been explored within the study's analytical framework, and all interview transcripts and fieldnotes have been explored through the lens of abjection. The diverse dental disgust components that have emerged have further been investigated as articulations of dental disgust. The analytical framework is thus used methodologically to explore the experiences, practices and consequences of dental disgust and to create knowledge about how it can be de‐silenced, distributed and handled across institutional levels in practice. During the individual interviews with the carers, they were asked to recount dental care situations in which they experienced discomfort, including how they managed these experiences. This has been done to elicit insights into their current opportunities for handling dental disgust and identify which collective and institutional support features can maintain both the carer's well‐being and the quality of dental care in the future. The carers were informed that dental disgust was a project finding that occurred on the anonymous workshop post‐its and asked whether they had had similar experiences. The interviewer is, therefore, co‐responsible for some of the articulations of dental disgust, asking about it specifically. This ethnographic involvement has been necessary to move beyond the inappropriateness, secrecy and solitude associated with dental disgust. All informants were selected by local municipal project managers based on their responsibilities regarding daily dental care. The ethnographic material was collected between October 2021 and November 2023 and later transcribed. Selected quotes and field notes have been translated into English for this article. The author conducted the first analysis of the material, which was then discussed with the entire research team.

## 4 Ethics

Dental disgust is a precarious finding, raising crucial ethical concerns. Its dissemination risks stigmatising the cared‐for older persons as disgusting and the carers as uncaring; therefore, ethics is part of the study's key argument. The study aims to puncture the inappropriateness and secrecy of dental disgust, and it insists that disgust is an inevitable part of care work, carefully and critically offering solutions for use in practice. By analysing articulations of dental disgust, the study further commits to reimagining better dental care situations in the future. Thus, the study has several practical ethical aims (Melonçon et al. 2020). Moreover, the use of abjection functions not only as an analytical framework but also as an ethical manoeuvre, as it removes the focus from the individual carers and cared‐for and concentrates on what is between them. On this basis, all informants have been anonymised using pseudonyms, and they will further appear exceedingly blank and un‐described, as all their recognisable characteristics and their care units have been left out. This includes their titles and professional backgrounds, which is why they are all described as carers. Similarly, the cared‐for older people mentioned in the interviews will remain vacuous. This complete blurring has been necessary, as several carers feared exposing older persons, as well as workplace repercussions because of their participation. The interviews emphasised that the carers felt comfortable sharing their experiences and had no fear of judgement, with the option to pause or withdraw if needed, though no one did so. The ethnographic material was stored on encrypted drives according to current GDPR rules, and the project was approved by the Danish Data Protection Agency (514‐0599/21‐3000) in May 2021 and the local Research Ethics Committee at the Faculty of Health and Medical Sciences (504‐0241/21‐5000) on 30 March 2021.

## 5 Disgust as Intimidation, Unfamiliarity and Dirtiness

For Agathe and nearly all the other carers included in the *Lifelong Oral Health* project, dental care is ‘*far worse than other care tasks*’. It provokes abjection more often and profoundly than, for instance, changing diapers, which the carers often compare it to. This can seem puzzling, as work involving human excrement may be more immediately associated with disgust. However, while talking calmly about diaper changing, stating ‘*It doesn't bother me at all’*, Agathe's speech flow became tense while talking about dental care. Like most carers, she frivolously began using sounds and exaggerated gestures to articulate the abjection it provoked for her. ‘*I get the reflex, you know. The* (deep sound of vomiting followed by an explosive arm movement from the mouth)*. Because it is right there, in your stomach. And up in your throat’*. Similar articulations of physical discomfort, for instance, encompassing gestures such as holding a hand in front of the mouth, were present in most individual interviews with carers, as were outbursts such as ‘*yuck*’, ‘*ouch*’ and ‘*whew*’. Therefore, the analysis will explore why dental care is particularly abjected. It adopts a multisensory approach to dental disgust, being inspired by recent sensory studies and anthropologist David Howes's concept of the sensorium, viewing sensory experiences as fluid intertwinings between mind, body and environment (Howes 2024; Wilkinson and Wilkinson 2020). The sensorium refers to the complete variety of sensory experiences as inseparable from the cultural, social and affective frameworks shaping it (Howes 2024). Howes's sensorium aids in understanding how sensory experiences relating to dental care are deeply intertwined with emotional experiences and how these are interpreted within the care system. Therefore, the following analysis does not simply explore dental disgust as a visceral reaction triggered by, for example, a smell, rather it understands dental disgust as deeply embedded in socio‐material everyday life in care institutions. The first analysis examines three recurring components of the carers' articulations of dental disgust: intimidation, unfamiliarity and dirtiness.

Dental care uniquely challenges the confines of the human intimate sphere. Articulations such as ‘*It is so intimidating’, ‘It is the most transgressive care task I have’ and ‘It is almost too close*’ recur in the interviews with the carers, revealing that caring intimacy can become an uncomfortable sense of intimidation, which is a central component of dental disgust. The closeness of faces, eyes and breath separate dental care from other care tasks, and the intimate sphere is even more broken as it takes place within the mouth, inside the threshold of the body. ‘*It's uncomfortable that you have to put your hand all the way in*’, the carer Liv said. Furthermore, intimidation and the breaking of bodily boundaries intensify sensuous experiences when encountering the mouth, making, for example, oral malodour more significant. ‘*It's the smells.* (…)*. They simply make everything so nauseating. And it is right up your face*’, the carer Simone said. The closeness also causes saliva and toothpaste to easily spray back onto the carers' faces, which means that ‘*You are always alert when you enter a mouth’*, as Simone stated. According to Kristeva, abjection can be triggered when ‘*An incandescent, unbearable limit between inside and outside (…) is crossed’ and when “the intimate” (…) topples over into “the nerves”’.* (1982, p. 190). In line with Kristeva's thoughts, these articulations of intimidation show that caring closeness can become unbearably intimidating, which largely forms part of dental disgust.

Despite its central placement in the face, the mouth can be an unacquainted and hard accessible territory of the body. A pervasive unfamiliarity with the mouth is another profound component of dental disgust when it is explored through the abjection lens. It is difficult to obtain a well‐lit view and a good working position that enables sufficient oral care, making the mouth challenging to navigate. ‘*Does it hurt? Am I doing it too hard?* (…) *Am I brushing all my teeth? Basically, I don't know’,* Liv said. In Denmark, carers do not receive mandatory dental care education, which underpins the unfamiliarity of the mouth. To the untrained eye, it can be difficult to tell whether oral disease is present or the teeth are simply aesthetically marred. Therefore, the carers often do not know what to look for and react to. This issue is further complicated due to the ‘dental transition’, which refers to the demographic changes, with more and more older people keeping their own teeth longer and therefore needing more and more complex dental care (Petersen et al. 2021). This emphasises that dental care is ‘*one of the most difficult tasks we have*’, as the carer Linda said. This unfamiliarity also shapes the sensuous experience of dental care. ‘*Well, we know how it smells on the other end. A fart smells like a fart, and a urinary tract infection smells like a urinary tract infection, but the mouth smells differently each time. It's just different with the mouth’*, the carer Dorthe said, indicating that the unfamiliarity of the mouth may make dental care evoke more abjection than diaper changing. Furthermore, it makes several carers afraid of putting the toothbrush too far down the throat, risking the older person vomiting. This is a concern for the older person but also for the carers themselves, as ‘*Vomit makes you feel bad yourself*’, as Pil said. The unfamiliarity of the mouth thus contributes uncertainty, unexpectedness, doubt and nervousness to dental disgust.

Another component of dental disgust is a sense of dirtiness, which is aligned with anthropologist Mary Douglas’s notion of dirt as ‘*matter out of place*’, as outlined in ‘Purity and Danger’ (Douglas 2002). Douglas explains that dirt is perceived not as an inherent feature but as contextual and relational. The ethnographic material shows that dental disgust often occurs when something related to the mouth is out of place, for example, the teeth themselves when they are loose, broken or missing or, especially, when food leftovers are present in and around the mouth. However, the experience is particularly evident when working with dentures. Douglas notes, ‘*What is clean in relation to one thing may be unclean in relation to another*’ (2002, p. 9). No carers describe a clean and fitted denture as abjected, but when it is loose or outside the mouth, it can become a source of abjection, especially if food leftovers are present as well. When describing the first time she rinsed a denture, the carer Aya said, ‘*Standing there with a denture, with lots of leftover food (…), of course, we wear gloves, but uhhhhh. I kind of sensed all the food leftovers right through them. Well, it was simply disgusting*’. The dirtiness of caring for mouths and dentures thus implies a significant contextual element of dental disgust.

Intimidation, unfamiliarity and dirtiness stand out as essential components of dental disgust when it is explored through the abjection lens. These components crucially shape dental care, making carers struggle to maintain it. For example, Agathe ‘*gets it over with*’ by constantly attempting to counteract the gag reflex, thinking to herself, *‘Hold it a little longer, just a little longer’* while completing the task in the shortest possible time. In other cases, the carers distance themselves from mouths and dentures when performing the care work to endure it. *‘I'm looking away from it* (dentures) *while I'm rinsing it.* (…) *I kind of distance myself from it,’* Aya said. Similarly, the carer Ilja said about toothbrushing, *‘Well, you shouldn't stand too close. You can do it from a little more distance. Nice and easy, certainly without looking directly in the mouth.* (…) *It is simply too intimate if I look directly in their mouth’.* Others turn to remedies to create a distance. ‘*I always make sure I wear gloves, and then, I use a foam cloth. I help click off the denture and then carry it on the cloth’,* Agathe said, while mentioning that the gloves are sufficient to maintain hygiene and prevent contamination. She only uses the cloth ‘*to protect myself’,* she said, referring to the discomfort the task provokes. In the same manner, Victoria turned to remedies to shield herself, *‘If it is too much, I put on a mask and take a mint’.* Thus, gaze direction, body placement and various remedies act as a diverse set of socio‐material articulations of dental disgust, decisively shaping how daily dental care is performed. Sometimes, these articulations enable dental care work. Yet, mainly, they hinder adequate dental care, as the carers' need for distance and time efficiency prevents thoroughness and makes them unable to detect oral diseases. Accordingly, one of the most widespread articulations of dental disgust is not practising dental care at all: ‘*I don't do toothbrushing*’, ‘*When it is too much, I skip it*’, ‘*I prioritise otherwise*’. Thus, several of the carers' diverse articulations of dental disgust pose substantial barriers to older peoples' vital, preventive and curative dental care.

## 6 Disgust as Shame and Inappropriateness

The carer's articulations of dental disgust often involve a confession. Their speech flow is frequently interrupted by pauses, sighing and self‐judging statements, such as *‘I'm not proud about it’, ‘I hate to admit it’ and ‘I feel so bad saying this’*, which are often expressed while hiding the face in the hands. ‘*I think it is so difficult to talk about. I don't feel I have the right to feel this way*’, the carer Pil said. These confessions reveal that dental disgust is often intertwined with shame. According to Kristeva, abjection appears when the subject fails to manage the corporal boundaries between the self and what is abjected. Therefore, abjection is deeply associated with shame (Kristeva 1982, 8). The philosopher Martha Nussbaum writes, in ‘Hiding from Humanity; Shame, Disgust and the Law’ (2009), that disgust and shame are related, as shame can arise from disgust, leading people to view themselves as inadequate and unworthy, which applies to many carers. As disgust and shame both concern bodily integrity and social norms, they can further harmfully reinforce one another, perpetuating social hierarchies and exclusion (Nussbaum 2004). This dynamic is highly at stake concerning dental disgust, making carers hide it. It is further complicated when carers have obvious oral health issues themselves, increasing the sensitivity of initiating and performing dental care. ‘*I don’t feel I can tell them to brush teeth when I look this way*’, the carer Henrik said to two members of the research team during an informal conversation in a project workshop break, ensuring others did not hear. In an earlier phase of the Lifelong Oral Health project, tooth shame (shame regarding teeth and oral health issues) was identified as a significant barrier to oral health, as it often spreads in and between the cared‐for older people and the carers, drawing everyone into sensitive shame situations, with serious implications for dental care practices (Folker et al. 2024). Many carers, therefore, avoid dental care out of thoughtful consideration for older persons, ensuring not to expose them. Thus, diverse components of shame also form part of dental disgust, catalysing myriads of self‐reinforcing sensitive processes, which greatly interfere with dental care situations.

The lens of abjection reveals that such dynamics are further sustained by work environments in which dental disgust is not openly discussed. When asked whether dental disgust had ever been a topic of conversation at the care unit, Agathe responded, ‘*No, never. Maybe you feel it in the air sometimes, but not more than that*’. Moreover, the interviews with local managers show they often do not recognise it. ‘*None of my employees find it disgusting. It takes more than that to knock them out*’, a manager said. Despite strong support for the employees, this reveals that dental disgust is difficult to spot for the managers, as it was highly prevalent in the unit. Additionally, managers and collaborative partners often direct frustration towards the carers when presented with the poor oral health status of the older people in their units, as revealed by the clinical registrations in the Lifelong Oral Health project. One manager pointed to laziness among the carers, saying ‘*I can't deny that some of them are cutting corners. Not everyone is equally committed*’. A managing dentist accused the carers of ‘*sheer indifference! They are simply careless with these older people*’, she said, obviously upset about prevalent oral hygiene issues in the municipality. Thus, the carers' intentions in avoiding dental care are often misunderstood, leading to collaborative conflicts and a tense atmosphere.

Dental disgust also leads to frustration between colleagues. When informed that dental disgust was a general project finding, the carer Victoria, irritated, said, ‘*I don't think you should be in care work if you feel disgusted. It annoys the hell out of me. We help people who can't help themselves. (…) It's unprofessional*’. Her reaction criticises not only the avoidance of dental care but also the very experience of disgust. However, she also openly talked about shielding herself with masks and mints during dental care, which she always carries in the pocket of her uniform. In sum, the frustration and blame among managers, collaborative partners and colleagues indicate that dental disgust is socially unacceptable in the care institutions. Within Haraway's line of thought, the disgusted carers become ‘*inappropriate/d others’,* which means being *‘in critical, deconstructive relationality, in a diffracting rather than reflecting (ratio)nality.* (…) *To be inappropriate/d is not to fit in the taxon, to be dislocated from the available maps specifying kinds of actors and kinds of narratives*’ (Haraway 1992). When the carers are caught in a diffracting, rather than a reflecting, rationality, they cannot express their experiences of dental disgust openly in their workplaces. The diffracting inappropriateness, which is sustained both externally by managers, collaborators and colleagues and internally by the carers themselves, deeply marginalises dental disgust, leaving the carers solely responsible for handling it. On this basis, it is crucial to explore their articulations further to clarify how the inappropriateness can be dissolved, and how dental disgust can leave from the secretive margins.

## 7 Reimagining the Future With Disgust

The carers' articulations of dental disgust consist of hopes for a better future. In between and integrated into the carers' shameful confessions, their articulations also include new ideas and solutions with which to improve dental care situations. The articulations reflect another key finding of the Lifelong Oral Health project: that dental care is remarkably absent in the Danish care system. Therefore, several of the carers' articulations emphasise making dental care a part of their professional foundation and ‘*part of the daily routine, so everyone can get used to toothbrushing, both carers and cared‐for’,* as Victoria said, while further reflecting that *‘it has to come from the management. Otherwise, it just falls flat again’*. This final part of the analysis will explore how the carers' articulations of dental disgust, also imply a hopeful reimagining of a better future for everyone involved in dental care. Many of the carers' specific ideas and solutions are highly locally grounded, reflecting the need for diverse future initiatives adjusted to particular care environments and requirements. Thus, this section presents the carers’ ideas in overall terms, which can then inspire more specific local adaptations. Especially, it zooms in on the carers' hopes for collective and institutional support features to back more robust dental care situations.

First, the carers articulate a hope for more education. ‘*It would have been totally different if we had some training, so we got used to it and knew what to be aware of*’, Aya said. Like several other carers, she noted that training would reduce the unfamiliarity of the mouth. This includes practical skills, such as working positions and appropriate tools and knowledge about oral diseases, symptoms and medical preparations. Some carers also articulate a hope for competence‐developing initiatives. ‘*It could be a bit like recurring first aid courses, where you keep your knowledge up to date and teach old dogs new tricks. (…) It could be a dental hygienist coming in to give a talk’,* Victoria said. Training stands out as important regarding disciplining disgust in general. During a focus group interview, a carer and a dental hygienist discussed training in relation to disgust experiences. The carer said, ‘*It's a bit peculiar, but when you're a healthcare professional, performing lower‐body hygiene is much less intimidating than brushing someone else's teeth*,’ to which the dental hygienist responded, ‘*Well, for me, changing a diaper is much more disgusting than brushing someone's teeth. I think our different educations shape our attitudes toward these tasks*’. In line with Kristeva's notion of abjection as inherently transformative regarding the subject's boundaries and identity, these quotes reveal that training can be a catalyst for such transformations since different professions can give shape to different abjections. With hopes for future training, the participants thus not only point to practical skills but also to professional identity. However, research within the field of odontology on emotional barriers to dental care demonstrates that education alone is insufficient, as additional knowledge alone does not change such barriers (Johnson 2013). As a supplement, several carers articulate a hope for ongoing peer‐to‐peer initiatives, including stronger collaboration between elderly care and dental care professionals. The experienced carer Linda, who was about to retire and who had been performing dental care throughout her working life and felt fine doing so, said, ‘*The old ones among us, such as myself, must have the time and opportunity to take the young carers by the hand and get them used to it from the beginning*’. The opportunity for practice training under the supervision of colleagues and collaborators is thus articulated as a potential future support feature.

Second, the carers articulate a hope for an institutional understanding that dental care requires time, calmness and the opportunity to build trusting care situations. Especially, they long for more time to ensure they do not amplify shame. As Øzlem puts it, ‘*When I know that the next person in the evening care routine has to take their medication at a fixed time, it's not possible to provide dental care in a calm and pleasant way that doesn't feel too exposing*’. In this way, she points to the resource‐related preconditions of (dental) care. In addition, several carers mention that dental care requires not only sufficient time for each situation but also an opportunity to get to know the cared‐for persons over time. ‘*It's almost only my own residents that I'm allowed to brush teeth on, but that's probably because we know each other so well. When I have to attend to someone else, they refuse. My own residents (…) tend to open their mouths more*’, Linda said, indicating that time to create personal relationships is crucial when disgust and shame interfere with dental care. Many carers articulate a hope for more time, including the institutional handling of busyness, turnover and understaffing, to mitigate dental disgust in the future. However, the fieldwork also points to complexity, as some carers describe the fact that dental care can be so intimidating that it can place a good caring relationship at risk and hinder other crucial care tasks. Despite this complexity, most carers articulate that time, calmness and trust can help dismantle dental disgust in the future.

Third, the carers articulate a hope for a more intuitive and accessible dental care infrastructure in formal systems. This includes digital health record systems, info screens, websites and analogue systems maintained on whiteboards, posters and paperwork. They especially point to the digital health record systems that stipulate the daily routines for each cared‐for older person. ‘*When dental care is stated in Cura* (a digital health record system)*, it becomes a task. Then, it is much less intimidating to actually do it, to brush other people's teeth. It's super unnatural to walk through someone's door and ask them if you should brush their teeth, but once it is registered, it's your job*’, the carer Pia said, indicating that a clear formal infrastructure has transformative potential regarding dental disgust. It is a recurring finding across carers and managers that they yearn for digital health record systems to be more accessible and intuitive regarding dental care. The carer Signe noted, ‘*It is just weird. In Nexus* (a health record system), *we have a tab on “diet”, “pressure ulcers” and “skin”, but we don't have a tab on “dental care.” We don't have a tab to support it. We must have that (…) and the dental unit should be able to access it as well. Today, we can't access the same systems*.’ Similarly, several carers hope for dental care to be more present at locally anchored systems, such as overview boards for each cared‐for older person used in the daily staff meetings. ‘*Maybe there could be a magnet with a toothbrush icon on our board to clarify who the dentist said needs assistance with dental care, to make it easier to hold on to’*, the carer Yasmin said. Thus, the carers articulate a hope for the highlighting of dental care in formal systems to make it a clearer part of future daily routines.

Finally, the carers articulate a hope for more trusting, supportive and secure work environments in which they can openly discuss the sensitive situations dental care implies without fearing judgement and workplace repercussions. They hope their institutions will encourage them when dental care becomes overwhelming, including support from central and local managers at both formal and informal meetings. ‘*It would help to have a more open conversation about it. When we do eventually talk about dental care in staff meetings, people kind of hide and cover themselves up. We know that it’s not being done, but no one openly states that they don't do it and don't feel comfortable with it. (…) We need to open up about this*’. Liv said, similarly to Victoria. ‘*I think we lack a working community around this’*. In addition, the retiring carer, Linda, hoped for more openness, as she frequently experiences younger carers and students asking her for help with diaper changing but rarely dental care, indicating that abjection regarding human excrement is backed by a higher social acceptance in the care settings. She, therefore, pointed to institutional initiatives regarding help‐seeking. Furthermore, the longing for openness was highlighted in the interviews, as the carers would often end with articulations of relief. ‘*Oh, I thought I was the only one who felt this way. Thank you so much for telling me this* [that other carers also experience dental disgust]’, Ilja said. Likewise, Aya shared, ‘*Well, toothbrushing is a difficult task, Louise* [interviewer and author]. *I'm so glad if you're telling our managers about our challenges because it can be hard for us to tell them ourselves*’, concluding the interview. ‘*Can I give you a hug now?*’ These hopeful and relieved articulations show that openly confronting dental disgust can transform it from a source of shame into a common and manageable care condition, enabling its deliberate and collective handling in the future.

## 8 Conclusion

This study has suggested an analytical framework for understanding care‐related disgust, which can act as inspiration in various care settings regarding various kinds of disgust. Drawing on Julia Kristeva's notion of abjection, it has provided an analytical lens via which to confront and transform disgust in praxis settings. By further incorporating Donna Haraway's thoughts on articulation as a means for social change, the study has further investigated how disgust is articulated in practice, including how it can be acknowledged and handled more compassionately and effectively in the future. Taking its point of departure in this analytical framework, the article itself acts as an articulation of dental disgust. Both due to its theoretical research contribution concerning care‐related disgust and due to its practical conclusions regarding the improvement of oral health in empirical care settings. It thus reimagines a future social change in care studies as well as care settings. When care is idealised as purely noble, the monstrous, brutal and yet vital aspects of care are wrapped in secrecy, shame and marginalisation, instead of being recognised as integral to caregiving. On this note, this study challenges previous research claims that care is a solution to abjection; instead, it asserts that future solutions entail an open and collective distribution of care‐related abjection. Concurrently, the article has demonstrated the importance of investigating disgust empirically through ethnographic methods to explore it as a socio‐materially situated phenomenon. This approach is crucial, as the components of disgust vary from praxis to praxis. On this basis, the article calls for future empirical research exploring other kinds of disgust in other socio‐material care settings to generate knowledge about its consequences and enabling potentials.

As its specific case, this study has identified and explored dental disgust as a distinct social phenomenon of the human mouth. It has found that dental disgust is a persistent everyday experience for carers in elderly care settings, comprising care‐hindering components of intimidation, unfamiliarity and dirtiness, with substantial implications for dental care situations and older peoples' oral health. The study's aim has not been to remove dental disgust totally but, rather, to suggest how it can be handled collectively. It notes that diaper changing, in contrast to dental care, has become normalised in care settings, even though students and newer caregivers often experience initial disgust. The difference is that diaper changing is institutionally supported through infrastructure, training, formal systems and open workplace conversations, whereas dental care lacks such backing. This study, therefore, advocates for institutional changes to support individual carers when dental disgust interferes with their caring obligations for the sake of both the carers' well‐being and that of those they care for. The reason this article does not provide specific recommendations on how to manage disgust in practice is that it is a finding that solutions vary from care setting to care setting. The article can thus be regarded as an inspiration for what to focus on when developing localised solutions. When public care institutions take responsibility for the vital dental care of older people, dental disgust becomes an institutional and research priority. Consequently, if the severe impairment of oral health among care‐dependent older people is to improve, carers must receive the support they need to confront and transcend dental disgust, creating opportunities for connections and compassion in future dental care situations.

This study calls for further research to examine the socio‐political dimensions of dental disgust, including whether and how it intersects with categories such as gender, sex, race, class, disability and general health status, including potential entanglements with self‐disgust (Gibson et al. 2004). Such research could address whether and how dental disgust places certain groups of people at a greater risk of receiving inadequate dental care than others. There are indications in this study that the gendered and racialised aspects of dental disgust are especially significant, as is well‐documented for other kinds of disgust (Wolf‐Meyer 2024; Hasan 2019), but the Lifelong Oral Health project does not have sufficient data to state this. It also does not have a sufficient ethical basis to comment on gender, race, socio‐economic life circumstances and so forth, as these data are not shared by the participants or are not part of the conditions they have or consented to. The study, however, strongly encourages such future research, which could take inspiration from Doughty et al.’s work on oral health‐related stigma (2023), and Muirhead et al.’s work on intersectionality concerning oral health issues (2020). Moreover, this study recommends examining dental disgust in other healthcare settings and social sectors. Additionally, it encourages broader social science studies on the mouth's social and cultural significance across life stages to illuminate its profound role in human life.

## Author Contributions

**Louise Folker:** conceptualization, resources, data curation, formal analysis, investigation, methodology, writing – original draft, writing – review and editing.

## Ethics Statement

This study was approved by the Danish Data Protection Agency (514‐0599/21‐3000) in May 2021 and the local Research Ethics Committee at the Faculty of Health and Medical Sciences (504‐0241/21‐5000) on 30th March 2021. The study was conducted in accordance with the American Anthropological Association Statement on Ethics (https://americananthro.org/about/policies/statement‐on‐ethics/).

## Consent

Informed consent was obtained from all individual participants included in the Lifelong Oral Health project. All individual participants also gave informed consent regarding publishing their data.

## Conflicts of Interest

The author declares no conflicts of interest.

## Data Availability

The data that support the findings of this study are not available nor published. All participating informants have been promised anonymity. It is impossible to anonymize the dataset completely.
